# The Prediction of Antioxidant Q-Markers for *Angelica dahurica* Based on the Dynamics Change in Chemical Compositions and Network Pharmacology

**DOI:** 10.3390/molecules28135248

**Published:** 2023-07-06

**Authors:** Hui Gao, Qian Li

**Affiliations:** State Key Laboratory of Aridland Crop Science, College of Agronomy, Gansu Agricultural University, Lanzhou 730070, China; huigao1210@163.com

**Keywords:** *Angelica dahurica*, GC-MS, antioxidant, network pharmacology, Q-Markers

## Abstract

Objective: To clarify the accumulation and mutual transformation patterns of the chemical components in *Angelica dahurica* (*A. dahurica*) and predict the quality markers (Q-Markers) of its antioxidant activity. Method: The types of and content changes in the chemical components in various parts of *A. dahurica* during different periods were analyzed by using gas chromatography-mass spectrometry technology (GC-MS). The antioxidant effect of the Q-Markers was predicted using network pharmacological networks, and molecular docking was used to verify the biological activity of the Q-Markers. Result: The differences in the content changes in the coumarin compounds in different parts were found by using GC-MS technology, with the relative content being the best in the root, followed by the leaves, and the least in the stems. The common components were used as potential Q-Markers for a network pharmacology analysis. The component-target-pathway-disease network was constructed. In the molecular docking, the Q-Markers had a good binding ability with the core target, reflecting better biological activity. Conclusions: The accumulation and mutual transformation patterns of the chemical components in different parts of *A. dahurica* were clarified. The predicted Q-Markers lay a material foundation for the establishment of quality standards and a quality evaluation.

## 1. Introduction

The herbal medicine known as *Angelica dahurica* (Hoffm.) Benth. & Hook.f. ex Franch. & Sav. is the dry root of *A. dahurica* (Fisch, ex Hoffm.) Benth.et Hook. f. or *A. dahurica* (Fisch. ex Hoffm.) Benth.et Hook. f. var. *formosana* (Boiss.) Shan et Yuan [1]. It was first documented in *Sheng Nong’s Herbal Classic* and listed as a middle grade. *Angelica dahurica* (*A. dahurica*) is a perennial herb and its entire growth period is three years. The year of planting is the seedling period and the second year is the vegetative growth period, which is harvested when the plant withers. The plants collecting seeds enter the reproductive growth period in the third year [2]. The vegetative growth stage of *A. dahurica* can be divided into four stages: the seedling stage from mid-September to early March of the next year, the leaf growth stage from early March to early May, the root growth stage from early May to mid-July, and the harvest stage from mid-July to mid-September [3]. At present, *A. dahurica* has only root medicine, but Wang et al. [4] found that, in addition to root medicine, there are also records of leaf medicine. The chemical compositions of its different medicinal parts are obviously different, and these chemical compositions and pharmacological activity change greatly in different periods [5]. Therefore, the various parts of *A. dahurica* were studied according to its different growth periods to clarify the accumulation and mutual transformation laws of the chemical components in these different parts.

The pharmacological effects of *A. dahurica* extracts include antioxidant [6], anti-inflammatory [7], immunoregulation [8], antitumor [9], and anti-Alzheimer [10] effects. In recent years, experts have used a variety of modern analytical techniques to conduct in-depth research on the pharmacological effects and chemical components of *A. dahurica* [11,12]. They have shown that [13,14] it mainly contains coumarin and volatile oil, as well as polysaccharides, glycosides, trace elements, and other effective ingredients. Network pharmacology, a research method that conforms to the multi-component and multi-target effects of traditional Chinese medicine, has emerged in recent years, expanding research directions and providing new ideas for the study of the mechanism of action of traditional Chinese medicine. It predicts potential targets as a whole and quickly and extensively screens them through a network analysis of the drug component target [15,16,17]. Due to the multi-component and multi-target characteristics of traditional Chinese medicine, detecting any of its components cannot represent its overall efficacy, and due to the complexity of traditional Chinese medicine, the quality control indicators of this traditional Chinese medicine have a low correlation with their effectiveness and a poor specificity. Therefore, Liu’s group [18] first proposed the concept of quality markers (Q-markers) in 2016, aiming to better control the quality of traditional Chinese medicine.

Therefore, this study used gas chromatography-mass spectrometry (GC-MS) technology to study the accumulation dynamics of the chemical components in different parts of *A. dahurica*, analyzing the differences in the types and contents of these chemical components in its roots, stems, and leaves at different stages, and clarifying the accumulation and mutual transformation laws of the chemical components in these different parts. Four common components of the different parts of *A. dahurica* from different periods were screened out, these common components were analyzed as Q-Markers for network pharmacology, and their targets of action and involved signaling pathways were searched. Finally, a “component-target- pathway-disease” network was established to explore the specific mechanism of *A. dahurica*’s antioxidant activity. Finally, the biological activity of the Q-Markers was verified through molecular docking technology.

## 2. Results and Discussion

### 2.1. Analysis of Chemical Constituents in Different Parts of A. dahurica

**Figure 1:**
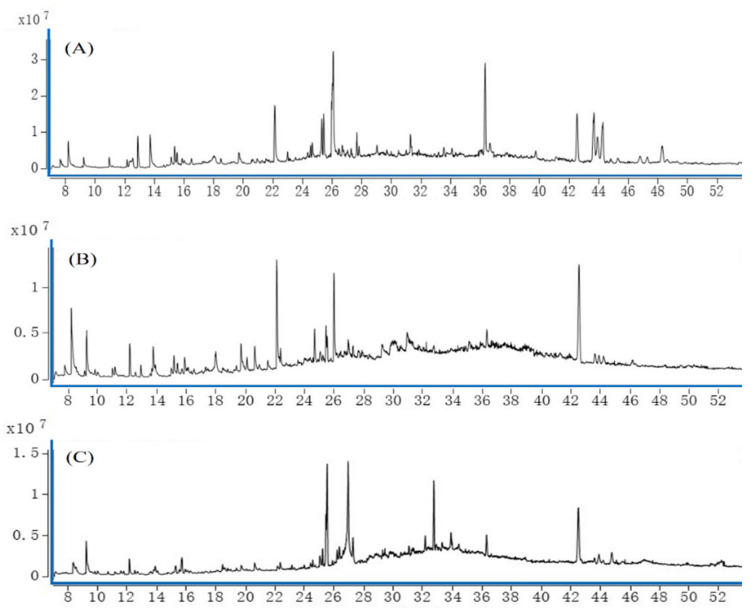
Total ion flow diagram of chemical components in *A. dahurica* at different parts. Notes: (**A**) root (**B**) stem (**C**) leaf.

Sample solutions of the roots, stems, and leaves of *A. dahurica* were analyzed using GC-MS technology to obtain the total ion flow diagrams of the mass spectrometry of its various parts. The total ion flow chart of the period can be compared with the highest accumulation of the compound content and types in each part, as shown in Figure 1. It can be concluded that, if the total ion flow diagram of each part is different, the type and content of the compound are different.

The chemical components and their relative contents in the roots, stems, and leaves of *A. dahurica* in different periods were obtained through the retrieval and analysis of the NIST14 standard mass spectrum library (Table 1). Sixty-one and forty-two compounds were identified in the roots and leaves in early September, and forty-eight compounds were identified in the stems in mid-July. Among them, the highest content in the roots was psoralen, the highest content in the stems was 5-hydroxymethylfurfural, and the highest content in the leaves was β-eudesmol. It can be seen that early September was the period with the most kinds of compounds in *A. dahurica*, and also the period with the most content accumulation.

### 2.2. Analysis of Main Chemical Components in Different Parts of A. dahurica at Different Stages

#### 2.2.1. Analysis of Chemical Components in the Roots of *A. dahurica* in Different Periods

In this study, imperatorin, oxypeucedanin, psoralen, and bergapten were used as representative coumarins to analyze the chemical components of *A. dahurica* at different stages. Imperatorin is the required analytical marker for *A. dahurica* stipulated in the *Pharmacopoeia of the People’s Republic of China*. According to the literature, the contents of oxypeucedanin, psoralen, and bergapten in *A. dahurica* are high and have very extensive pharmacological activities. Therefore, the four coumarins were selected for a subsequent chemical composition analysis.

**Figure 2:**
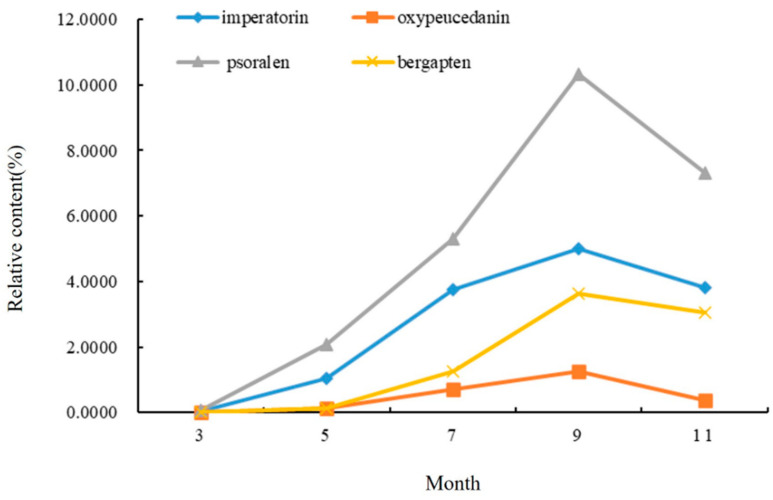
Changes in chemical components in roots of *A. dahurica* in different periods.

The relative content of the compounds in the roots of *A. dahurica* in different periods was analyzed using the peak area normalization method, and an accumulation dynamic analysis of the four important, effective components in the roots was carried out (Figure 2). As an index component of *A. dahurica* stipulated in *the Pharmacopoeia of the People’s Republic of China*, the relative content of imperatorin gradually increased from the seedling stage in March to the harvest stage in September, and rapidly increased from early May to mid-July. The relative content reached a maximum of 4.9971% in early September and began to decrease in November. Among the four components in the same period, psoralen had the highest relative content, reaching 10.3183% in early September. The relative content of oxypeucedanin was the lowest, followed by bergapten.

The relative content of coumarin in the root of *A. dahurica* in different periods increased first and then decreased. During the period of vegetative growth, from early March to early September, the coumarin content in root gradually increased, and the coumarin compound content reached its maximum in early September. After November, it entered the reproductive growth period. In order to maintain its life metabolism, the root system consumed some dry matter, so the relative content of coumarin showed a downward trend. This period was not conducive to an improvement in the quality of *A. dahurica*. To sum up, the coumarin content of the roots in the second year of growth showed an “S” trend.

#### 2.2.2. Analysis of Chemical Components in the Stems of *A. dahurica* in Different Periods

**Figure 3:**
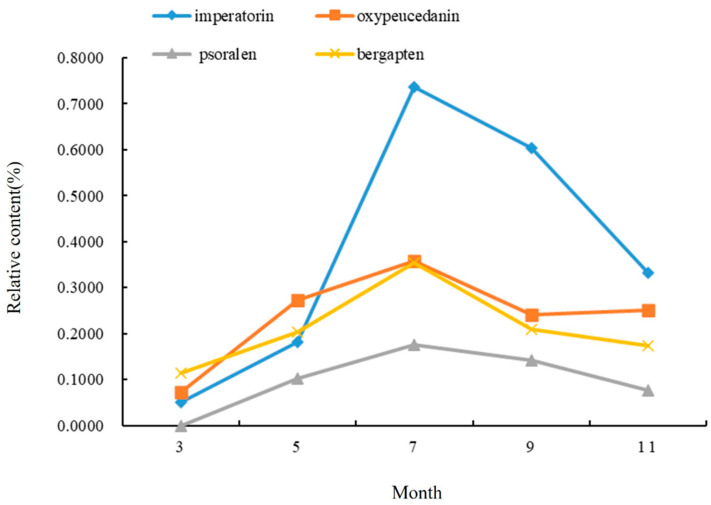
Changes in chemical components in stems of *A. dahurica* in different periods.

It can be seen from Figure 3 that the change in the coumarins in the stems of *A. dahurica* in different periods first increased and then decreased. From early March to mid-July, the aerial part was in a rapid growth period, so the relative content of the four coumarins gradually rose, and this content reached its maximum in mid-July. The highest relative content was imperatorin (0.7366%), followed by oxypeucedanin and bergapten, and the lowest content was psoralen, which is different from the results in the roots. After July, the contents of imperatorin, psoralen, and bergapten all showed a gradual downward trend, but oxypeucedanin began to rise again in September, and the total relative content was lower than that in the middle of July.

To sum up, mid-July is the period for the greatest accumulation of chemical compounds in the stems of *A. dahurica*. This indicated that coumarin compounds began to accumulate in the stems and transported less to the roots during the long period of leaf growth (from early March to early May). However, during root growth (from early May to mid-July), the coumarin compounds in the stems began to be transported to the underground part, and their own synthesis rate slowed down. In the harvest period especially (from mid-July to mid-September), a large amount of effective ingredients accumulated in the roots to promote the yield formation and improve the quality, which led to a decrease in the coumarin compounds content and species in the stems in early September.

#### 2.2.3. Analysis of Chemical Components in the Leaves of *A. dahurica* in Different Periods

**Figure 4:**
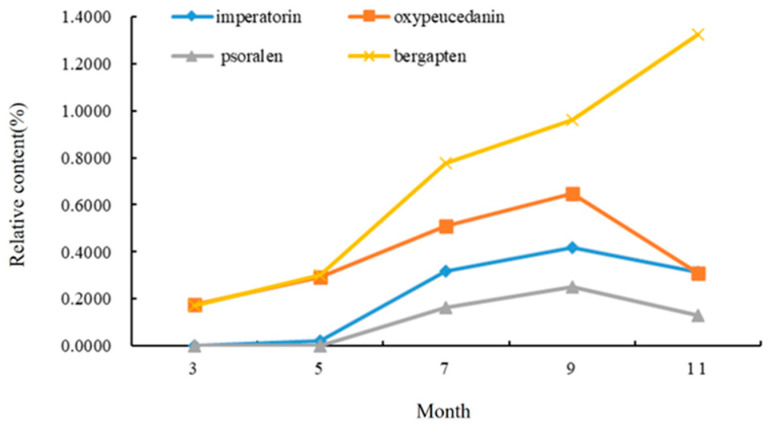
Changes in chemical components in leaves of *A. dahurica* in different periods.

It can be seen from Figure 4 that the relative content of the four coumarins gradually increased from early March to early September. The contents of imperatorin, oxypeucedanin, and psoralen reached the highest in early September, and the content of coumarin in the leaves decreased with the decrease in temperature in early November, but bergapten still increased gradually in November. The content of imperatorin increased rapidly in early September, reaching 0.4191%. The highest content of the four coumarins was bergapten (0.9598%), followed by imperatorin and oxypeucedanin, and the lowest content was psoralen (0.2499%).

To sum up, the total content of the chemical components in the *A. dahurica* leaves reached the highest in early September. The content of the coumarin compounds increased the fastest in the leaf growth period (from early March to early May), indicating that, with an increase in leaf area, more and more photosynthetic products were produced, and the coumarin compounds produced by the leaves increased rapidly. However, when the root growth rate increased, the leaves began to show a gradual aging trend, and the growth rate of the coumarin content in the leaves gradually decreased.

#### 2.2.4. Analysis of the Difference in Compound Accumulation in Different Medicinal Parts of *A. dahurica*

Table 2 shows the number of compounds identified in the different medicinal parts of *A. dahurica* at different stages. The results showed that the number of chemical compounds in the roots, stems, and leaves presented an “S” type change. In early March, there were more compounds in the stems than in the roots and leaves. From early May, the types of the compounds in the roots began to increase rapidly, exceeding those in the stems and leaves. The accumulation of compounds in the roots, stems, and leaves showed different trends. In the early September, the number of compounds in the roots and leaves was the largest, while in mid-July, the number of compounds in the stems was the largest. To sum up, most kinds of compounds were in the roots, followed by the stems and leaves.

**Figure 5:**
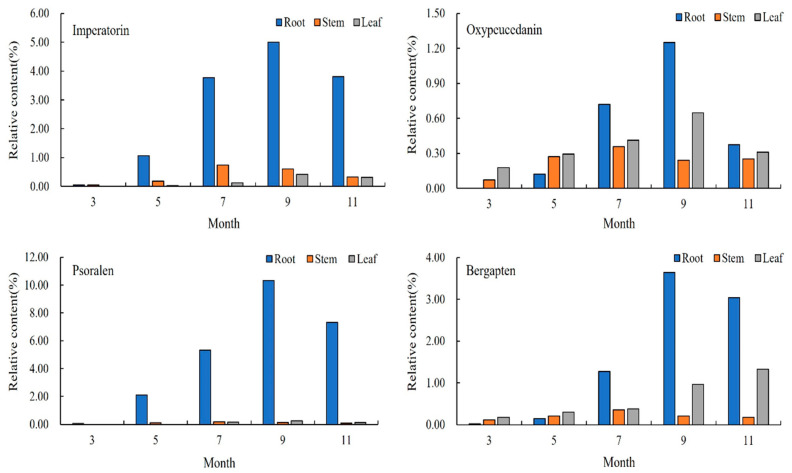
Changes in chemical components in *A. dahurica* in different parts at different periods.

The content changes in the four representative coumarins in the different parts of *A. dahurica* in the same period can be compared, as shown in Figure 5. In early March, imperatorin only existed in the roots and stems, and its content in the stems was higher than that in the roots. In early May, its content in the roots increased rapidly, and imperatorin also appeared in the leaves. From early May to early November, the relative content of imperatorin was maintained as root > stem > leaf. In early March, oxypeucedanin only existed in the stems and leaves, and in early May, it appeared in the roots, and the relative content was leaf > stem > root. In mid-July, the content of oxypeucedanin in the roots increased rapidly, and the content was higher than that in the stems and leaves, reaching the highest in early September. Psoralen only existed in the roots in early March, appeared in the stems in early May, and appeared in the leaves in mid-July, but its content in the roots was the highest, and its content in the stems and leaves was low. Its content in the leaves was higher than that in the stems from early September to November. In early March, bergapten was present in the roots, stems, and leaves, and the content of bergapten was maintained in the order of leaves > stems > roots from March to May. In mid-July, the content of bergapten in the roots began to increase, and after that, the content remained as root > leaf > stem. To sum up, the content relationship of the different parts of *A. dahurica* was: root > leaf > stem, indicating that the accumulation of coumarin in *A. dahurica* in the late growth period is mainly in the root.

### 2.3. Network Pharmacological Analysis

#### 2.3.1. The Potential Q-Markers of *A. dahurica*

**Figure 6:**
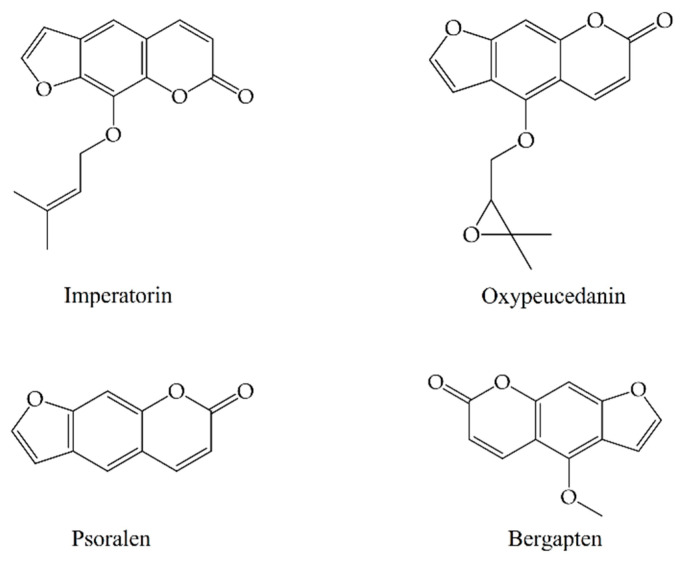
Structures of Q-Markers of *A. dahurica*.

A total of 61 chemical components of *A. dahurica* were retrieved using a GC-MS database. Based on the common components of its various parts at different stages, four compounds were ultimately selected as the Q-Markers of *A. dahurica*, including imperatorin, oxypeucedanin, psoralen, and bergapten. Their structures are shown in Figure 6.

#### 2.3.2. Q-Markers Targets and Antioxidant Targets

**Figure 7:**
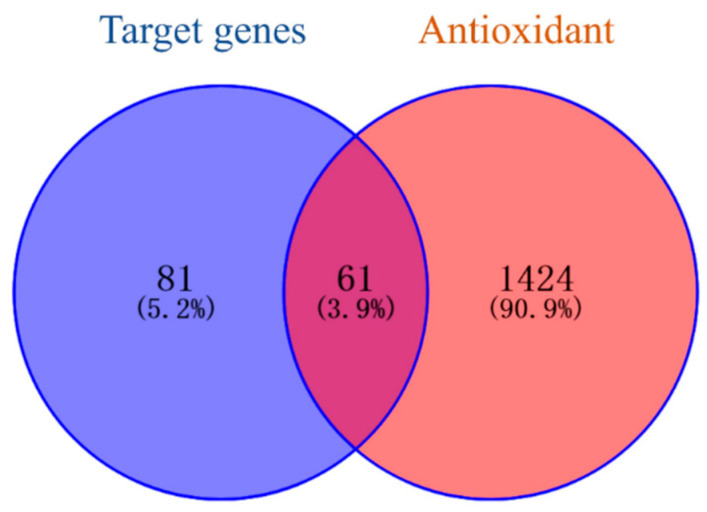
Potential antioxidant targets of Q-Markers in *A. dahurica*.

The Q-Markers obtained using GC-MS were searched through the TCMSP database and predicted based on the Swiss Target Prediction database platform. The Gene Names of the target genes were collected using the Uniprot database and duplicate or invalid targets were deleted. A total of 81 target genes of the Q-Markers in *A. dahurica* were obtained. A total of 1424 antioxidant-related targets were searched and screened in the Genecards and OMIM databases. The active ingredient targets and disease targets were mapped (Figure 7), resulting in 61 intersecting targets, which are potential antioxidant targets of *A. dahurica* (Table 3).

#### 2.3.3. PPI Network of Potential Antioxidant Targets of *A. dahurica*

**Figure 8:**
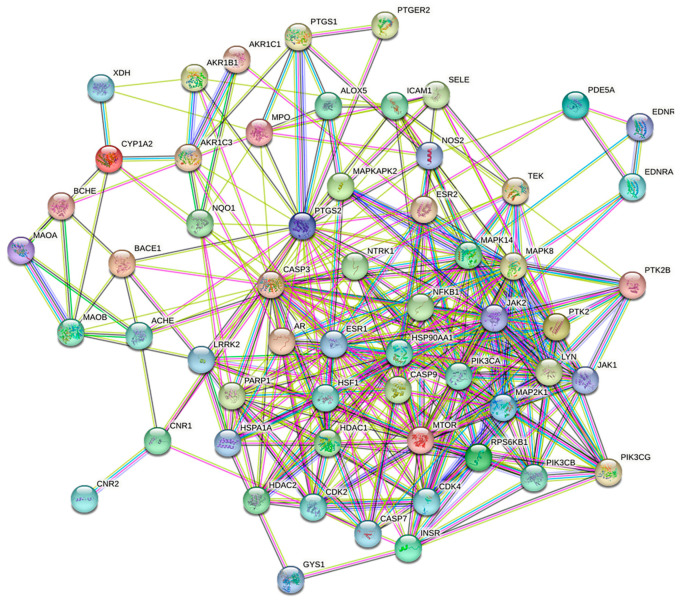
Target protein PPI network for antioxidant activity of Q-Markers in *A. dahurica*.

A total of 61 intersecting targets were obtained by intersecting the Q-Markers of *A. dahurica* with potential antioxidant targets. Then, a PPI network was created using these intersecting targets. As shown in Figure 8, the PPI had a total of 60 nodes and 353 edges, with 2 potential targets not involved in the protein interactions (PTAFR and PPOX). These two potential targets were removed.

#### 2.3.4. Enrichment of Antioxidant GO Function and Analysis of KEGG Pathway in *A. dahurica*

**Figure 9:**
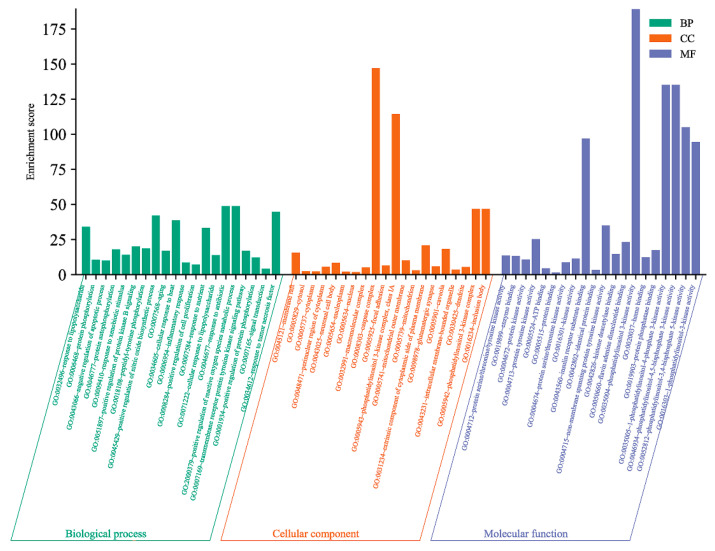
Analysis of antioxidant in Q-Markers of *A. dahurica* by GO Functional Enrichment.

The GO functional enrichment was analyzed through a database and it could be seen that there were 245 biological processes (BP) involved in the potential antioxidant targets of *A. dahurica*, mainly including its response to lipopolysaccharide, protein phosphorylation, the negative regulation of the apoptotic process, protein autophosphorylation, and its response to xenobiotic stimuli, etc. There were a total of 35 cellular components (CC), mainly involving the membrane raft, cytosol, cytoplasm, perinuclear region of the cytoplasm, and neuronal cell bod, etc. There were a total of 84 molecular functions (MF), including the protein series/threonine/tyrosine kinase activity, enzyme binding, protein kinase activity, ATP binding, and protein binding, etc. Among the three, *p* < 0.05 was selected and the top 20 counts were used for the GO functional enrichment map (Figure 9).

**Figure 10:**
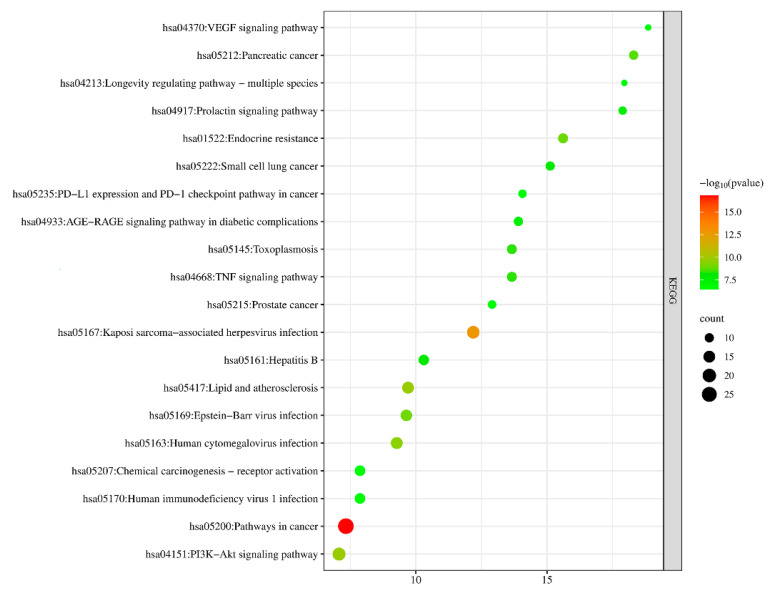
Enrichment analysis of antioxidant KEGG pathway in Q-Markers of *A. dahurica*.

The KEGG pathway enrichment was analyzed and the results showed that the potential targets of the antioxidant effects of *A. dahurica* mainly involved pathways such as the pathways in cancer, Kaposi-sarcoma-associated herpesvirus infection, lipid and atherosclerosis, human cytomegalovirus infection, and the PI3K-Akt signaling pathway, etc. *p* < 0.05 was selected and the top 20 counts were used for the KEGG pathway map (Figure 10). The KEGG pathway was consistent with the pharmacological activities of *A. dahurica*, including its antioxidant, anti-inflammatory, and anti-tumor effects, proving that it could effectively predict the pharmacological effects.

#### 2.3.5. Molecular Docking

**Figure 11:**
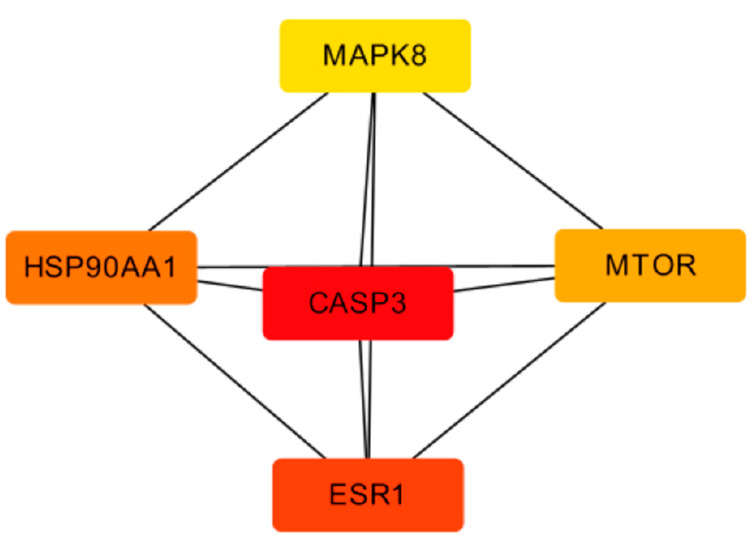
Core targets map of TOP 5.

The top five core targets were selected from the PPI network using the MCC algorithm and the Cytohubba plugin, as shown in Figure 11.

**Figure 12:**
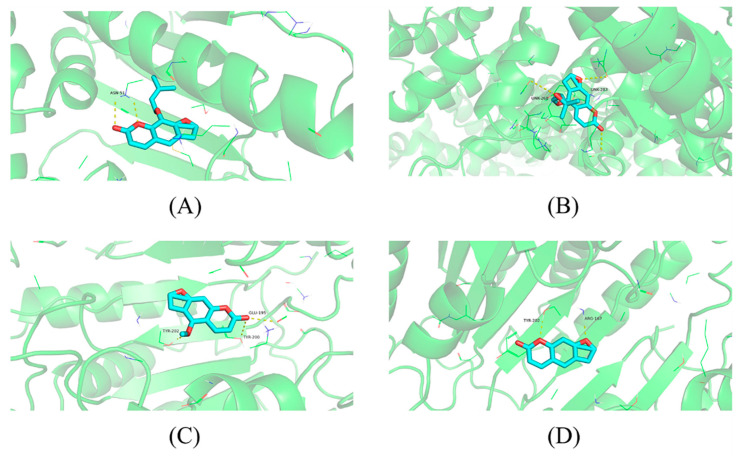
Molecular docking results. Notes: (**A**) Imperatorin and HSP90AA1, (**B**) Oxypeucedanin and MTOR, (**C**) Psoralen and CASP3, and (**D**) Bergapten and ESR1.

The results of the molecular docking between the five core targets and Q-Markers are shown in Table 4. The binding energy represents the advantages and disadvantages of small molecules binding to the target proteins, and a binding energy of less than 0 indicated that small molecules could freely bind to these target proteins. The lower the binding energy, the higher the likelihood of this binding. The Q-Markers had a good combination ability with HSP90AA1, MTOR, CASP3, and ESR1 on the key targets, indicating that the predicted Q-Markers of *A. dahurica* has good biological activity. In this study, the docking results of imperatorin and HSP90AA1, oxypeucedanin and MTOR, psoralen and CASP3, and bergapten and ESR1, with good docking results, were selected for display (Figure 12).

The key antioxidant targets of *A. dahurica* include CASP3, ESR1, HSP90AA1, MTOR, and MAPK8. The Caspase family is a convergence point of multiple apoptotic pathways [19], among which Caspase 3 mainly plays a role in executing apoptosis and is closely related to cell apoptosis. It can affect the proliferation and apoptosis of tumor cells through various mechanisms of action [20,21]. ESR1 belongs to endocrine hormones and research has shown that the skin itself has endocrine and immune functions, which can regulate each other [22,23,24]. The gene of HSP90AA1 (commonly known as HSP90) is situated on the chromosomes 14q32.2 [25]. In current cancer therapy, HSP90 is the center of attraction for its ability to inhibit multiple signaling pathways simultaneously [26]. HSP90 has been highly expressed in multiple cancers, such as lung, ovarian, endometrial, and pancreatic cancer, and additionally oropharyngeal squamous cell carcinoma (OSCC) and various myeloma [27]. The latest study revealed that a high HSP90 expression was a poor prognosis marker in different cancers such as lung cancer, melanoma, esophageal cancer, bladder cancer, and leukemia [28]. MTOR is a hub for various important signaling pathways within cells, regulating translation initiation, transcription, protein synthesis, and degradation functions, as well as important physiological functions such as cell survival, proliferation, and apoptosis [29,30]. The MAPK pathway is involved in processes such as cell differentiation, migration, proliferation, apoptosis, and inflammation [31,32]. It can be speculated that the Q-Markers of *A. dahurica*, including imperatorin, oxypeucedanin, psoralen, and bergapten, can exert anti-inflammatory, immune, and oxidative effects by regulating target proteins such as MAPK1, MAPK8, ESR1, and ESR2.

## 3. Materials and Methods

### 3.1. Materials and Reagents

The plant materials of *A. dahurica* used in this experiment were all fresh materials from Wufeng Town (Yichang, China) from March (seedling stage), May (leaf-growing stage), July (root growth stage), September (harvest stage), and November (reproductive stage) in 2021, which were identified by Professor Yuan Chen (Department of Chinese Herbal Medicine, Gansu Agricultural University, Lanzhou, China). The voucher specimens (No. GAUAB-AD-20210310, No. GAUAB-AD-20210507, No. GAUAB-AD-20210716, No. GAUAB-AD-20210905, and No. GAUAB-AD-20211104) were deposited in the herbarium of the Department of Chinese herbal medicine, Agronomy building of Gansu Agricultural University, Lanzhou, China. The methanol (Lot 67-56-1, Chromatographic grade) was purchased from Tianjin Beichen Fangzheng Reagent Co., Ltd. (Tianjin, China).

### 3.2. Analyzing the Main Chemical Components in Various Parts of A. dahurica at Different Periods by GC-MS

#### 3.2.1. Sample Solution Preparation

The *A. dahurica* in different periods was dried at 37 °C [33] and then crushed and sieved through 40 mesh. The medicinal powder of *A. dahurica* in different periods was weighed to 3 g, added to 30 mL of methanol in a conical flask, soaked for 30 min, extracted in a 40 °C water bath using ultrasound (power: 360 W, frequency: 40 KHz) for 30 min, cooled to room temperature, and then methanol was used to make up the lost weight. A certain amount of solution was taken into a centrifuge tube and centrifuged into a high-speed centrifuge (speed: 8000 r/min, time: 10 min), and then the supernatant was taken for 0.22 µm microporous membrane filtration to obtain the test solution.

#### 3.2.2. GC-MS Conditions

The data were obtained using gas chromatography coupled with triple quadrupole mass spectrometry (Agilent 7890B-7000D, Agilent Technologies Co. Ltd., Palo Alto, CA, USA). GC-MS column: DB-23 (30 × 0.25 mm × 0.25 μm), the carrier gas was high-purity helium and its flow rate was 1.0 mL/min, the inlet temperature was 250 °C, the initial column temperature was 35 °C, and this was maintained for 7 min. The temperature was raised to 280 °C at the rate of 10 °C/min and the samples were incubated for 5 min. The split injection was 10:1 [34].

The mass spectrum conditions: the electron impact (EI) ion source, full scanning mode (mass range *m*/*z* 30–500), ion source temperature of 230 °C; interface temperature of 250 °C; quadrupole temperature of 150 °C; electronic energy of 70 eV; solvent delay time of 3 min; and the ion detection mode (SIM) was selected.

#### 3.2.3. Data Analysis

GC-MS was used to conduct total ion scanning on the compound to obtain the total ion flow diagram of *A. dahurica* in different periods for its different parts. Through an analysis of the retention time and mass spectrum data of the compound, the obtained mass spectrum diagram was retrieved using the NIST14 standard mass spectrum library, and the chromatographic peak with a matching degree higher than 80% was selected for the qualitative identification of the compound. At the same time, the relative content of each component was calculated according to the peak area normalization method [35].

### 3.3. Prediction and Analysis of Antioxidant Related Substances in A. dahurica Based on Network Pharmacology

#### 3.3.1. Obtaining the Q-Markers of *A. dahurica*

Four common components were identified in each period and part based on the chemical compositions of the various parts of *A. dahurica* during the different periods measured using GC-MS technology. The Q-Markers in *A. dahurica* were further selected as active ingredients, and a network pharmacology analysis was conducted on the antioxidant effect.

#### 3.3.2. Screening of Q-Markers Targets

The selected Q-Markers were queried for Molecule Name in the TCMSP database (https://tcmsp-e.com/, accessed on 11 May 2023). Subsequently, the 3D structure and SMILES format of the compounds were obtained from the PubChem database (https://pubchem.ncbi.nlm.nih.gov/, accessed on 11 May 2023), imported into the Swiss Target Prediction database (http://www.swisstargetprediction.ch/, accessed on 11 May 2023), and analyzed by clicking on “Predict targets” to output the compound target information. The obtained compound target information was imported into the UniProt database (https://www.uniprot.org/, accessed on 14 May 2023), the species “Homo sapiens” was selected, and Probability > 0 was used as the screening criterion to obtain the corresponding predicted target.

#### 3.3.3. Screening of Potential Targets for Antioxidant Activity

The keyword “antioxidant” was entered into the GeneCards (https://www.genecards.org/, accessed on 15 May 2023) and OMIM databases (https://omim.org/, accessed on 16 May 2023) to search for the genes related to antioxidant activity in the database. The GeneCards database was screened using the median screening method to obtain score scores. All the antioxidant targets in the OMIM database were obtained, and then the duplicates were removed to obtain the disease targets related to antioxidant effects.

#### 3.3.4. Protein Interaction Network Construction (PPI)

The intersection of the Q-Marker targets and potential antioxidant targets was taken, and a Venn diagram was drawn using the online website Venny 2.1.0 (https://bioinfogp.cnb.csic.es/tools/venny/index.html, accessed on 21 May 2023). The intersection target obtained was the potential target of *A. dahurica*’s antioxidant effect. The potential target of *A. dahurica*’s antioxidant effect was imported into the STRING database (https://cn.string-db.org/, accessed on 21 May 2023) to preliminarily obtain the protein interaction network of the antioxidant effect, and the relevant data were exported. A protein interaction network was constructed for the antioxidant target using the String database (https://cn.string-db.org/, accessed on 21 May 2023).

#### 3.3.5. GO Enrichment Analysis and KEGG Pathway Analysis

The obtained anti-oxidation core target protein of *A. dahurica* was imported into the DAVID database (https://david.ncifcrf.gov/, accessed on 23 May 2023), and a Gene Ontology (GO) enrichment analysis and Kyoto encyclopedia of genes and genomes (KEGG) enrichment analysis were obtained. Entries with *p*-values of <0.05 were selected as significantly enriched GO entries or KEGG pathways.

#### 3.3.6. Molecular Docking

According to the degree values, the top 5 core targets were selected as key targets for the molecular docking visualization with the Q-Markers. Firstly, the SDF format file of the Q-Markers’ 2D structure was downloaded from the PubChem database, and the PDB format file of the core target protein 3D structure was downloaded from the RCSB database (https://www.rcsb.org/, accessed on 25 May 2023). Further processing of the protein and component data, such as hydrogenation and dehydration, was performed using Autodock Tools 1.5.6, which was saved in pdbqt format, and the binding energy was calculated.

## 4. Conclusions

In this study, the dynamic accumulation of the chemical components in different parts of *A. dahurica* at different stages was first investigated by using GC-MS technology. A total of 61 compounds were identified in *A. dahurica*, and the types and contents of the chemical components in each part were clarified. The relationship between the number and content of species in the roots, stems, and leaves was different. It could be found that, in the early stage, the stem and leaf growth was the main growth, while in the later stage, the root growth was the main growth. There was a clear competitive relationship between the aboveground and underground parts. Therefore, appropriate measures should be taken to control the growth of the aboveground part of *A. dahurica* after it enters the root growth peak period, so as to smoothly transfer the growth center from aboveground to underground and improve the quality of the medicinal material when using roots as medicine. The results of the network pharmacology research further explored the mechanism of the antioxidant effect of *A. dahurica*, indicating that this process involves multiple active ingredients, targets, and signaling pathways. Its mechanism of action is complex and diverse, and it is not only achieved through a single component, target, or the regulation of a single pathway, which is consistent with the multi-target characteristics of traditional Chinese medicine in treating diseases. The results of the macromolecular docking test showed that the active components of *A. dahurica* had a good binding ability with the core target related to antioxidants. In future studies, the spatial distribution of the compounds in different parts of *Angelica dahurica* at different stages and an experimental verification of the network pharmacology results could be carried out.

## Figures and Tables

**Table 1:** Analysis of chemical constituents of *A. dahurica*.

No.	RT	Compound	Molecular Formula	Relative Content (%)		
				Root	Leaf	Stem
1	10.730	Hexanal	C_6_H_12_O	0.0630	0.0458	0.4852
2	11.065	Phenyl α-D-glucopyranoside	C_12_H_16_O_6_	0.0618	0.2091	-
3	11.175	1-Heptanol	C_7_H_16_O	0.3544	1.1326	0.1893
4	11.735	Thymol	C_10_H_14_O	0.0870	0.1836	0.2834
5	12.467	3-Carene	C_10_H_16_	4.6076	0.2972	0.2763
6	12.930	3-Furaldehyde	C_5_H_4_O_2_	2.7352	0.4450	0.1074
7	13.216	Camphene	C_19_H_16_	0.0574	0.0574	-
8	13.770	3-Furanmethanol	C_5_H_6_O_2_	3.2033	2.2195	0.4839
9	14.032	(1S)-(-)-β-Pinene	C_10_H_16_	1.1364	0.0125	0.4001
10	15.056	2-Butenoicacid,2-methyl-, (Z)-	C_5_H_8_O_2_	2.7235	0.4160	0.5282
11	15.117	3-Methylcrotonaldehyde	C_5_H_8_O	3.4739	1.5817	-
12	15.410	2,6-Dimethylocta-2,7-dien-6-ol	C_10_H_18_O	1.7658	0.2394	1.1417
13	15.562	α-Phellandrene	C_10_H_16_	1.0273	0.4106	0.3924
14	16.884	4-Isopropylidene-1-methylcyclo	C_10_H_16_	0.8403	-	0.1160
15	17.774	Borneol	C_10_H_18_O	0.3705	0.1086	0.2096
16	19.718	4-Hydroxy-2,5-dimethyl-3(2H) furanone	C_6_H_8_O_3_	1.5613	-	0.8221
17	20.382	Acetone Glucose	C_9_H_16_O_6_	0.0652	0.2013	0.0567
18	21.503	4,6-O-Ethylidene-α-D-glucose	C_8_H_14_O_6_	0.9665	0.3251	-
19	22.356	Isopimpinellin	C_13_H_10_O_5_	0.5048	1.1532	0.9114
20	23.014	Isolongifolene	C_15_H_24_	0.8128	-	0.0789
21	23.051	Isobornyl acetate	C_12_H_20_O_2_	1.2771	-	-
22	23.143	Nonanal	C_9_H_18_O_7_	0.0889	0.6421	0.3161
23	24.197	Pimpinellin	C_13_H_10_O_5_	0.0828	0.2033	-
24	25.444	2-Hydroxy-5-methyl acetophenone	C_9_H_10_O_2_	3.5660	4.2425	-
25	25.988	5-Hydroxymethylfurfural	C_6_H_6_O_3_	3.6589	4.8772	0.3453
26	26.415	9-Hexadecenoic acid, (9Z)-	C_16_H_30_O_2_	1.1248	-	1.3593
27	26.805	Vetiverol	C_15_H_26_O	0.3599	-	-
28	26.823	β-Eudesmol	C_15_H_26_O	0.2134	1.0981	14.5071
29	27.055	Angenomalin	C_14_H_12_O_3_	7.5490	0.7771	-
30	27.085	Bisabolene	C_15_H_24_	2.0434	0.6263	0.6706
31	28.402	Paeonol	C_9_H_10_O_3_	0.8024	0.4961	0.9929
32	29.005	Acrylic acid tetradecyl ester	C_17_H_32_O_2_	0.7908	-	0.1187
33	29.249	Bergapten	C_12_H_8_O_4_	3.6443	0.3544	0.9598
34	30.126	Phellopterin	C_17_H_16_O_5_	0.2227	0.5170	0.2085
35	30.797	1-Hexadecene	C_16_H_32_	0.1534	0.3363	0.3949
36	30.803	Phenyl stearate	C_24_H_40_O_2_	0.0939	2.4649	-
37	31.376	Osthole	C_15_H_16_O_3_	1.4983	0.9714	0.6746
38	32.065	(+)-Decursinol	C_14_H_14_O_4_	1.1992	0.5716	1.5293
39	33.619	Imperatorin	C_16_H_14_O_4_	4.9971	0.7366	0.4191
40	33.997	2-Octylcyclopropaneoctanal	C_19_H_36_O	0.2201	0.2357	-
41	34.198	Pabulenol	C_16_H_14_O_5_	0.4830	0.2332	0.1453
42	35.417	Isooxypeucedanin	C_16_H_14_O_5_	0.2401	0.4490	0.2430
43	35.581	Isoimperatorin	C_16_H_14_O_4_	0.2248	0.2321	-
44	36.325	Methyl hexadecanoate	C_17_H_34_O_2_	1.0761	1.0578	5.0586
45	36.520	11-Dodecen-1-ol acetate	C_14_H_26_O_2_	0.0066	-	0.1124
46	36.648	Oxypeucedanin	C_16_H_14_O_5_	1.2500	0.3575	0.6478
47	38.184	Byakangelicol	C_17_H_16_O_6_	0.2069	0.2795	0.1002
48	40.231	Psoralen	C_11_H_6_O_3_	10.3183	0.1749	0.2499
49	40.445	Angelicin	C_11_H_6_O_3_	0.4101	-	-
50	41.798	13-Octadecenal, (Z)-	C_18_H_34_O	0.1218	0.0844	-
51	41.840	Verbenalin	C_17_H_24_O	0.0512	-	0.1104
52	42.023	1,2-Benzenedicarboxylic acid	C_16_H_22_O_4_	0.0543	-	-
53	42.096	Byakangelicin	C_17_H_18_O_7_	0.1309	0.0391	-
54	42.535	Dibutyl phthalate	C_16_H_22_O_4_	1.8615	-	-
55	43.669	Methyl oleate	C_19_H_36_O_2_	0.6757	0.7178	0.7278
56	43.894	Decursin	C_19_H_20_O_5_	3.8667	2.5107	1.4460
57	44.224	Methyl Stearate	C_19_H_38_O_2_	0.7647	0.3617	1.0879
58	45.735	Meranzin	C_15_H_16_O_4_	0.0411	0.3364	-
59	46.704	Columbianadin	C_19_H_20_O_5_	1.0762	0.1896	0.4628
60	48.983	1,4-Benzenedicarboxaldehyde,2,5-bis(hexyloxy)-	C_20_H_30_O_4_	0.1152	-	0.1347
61	50.141	Paullinic acid	C_20_H_38_O_2_	0.0291	-	-

**Table 2:** Number of compounds in *A. dahurica* in different parts at different periods.

Part	March	May	July	September	November
Root	22	42	54	61	49
Leaf	24	38	48	41	30
Stem	20	34	39	42	35

**Table 3:** Cross-target prediction of Q-Markers and diseases.

NO.	Target	Common Name	Uniprot ID
1	Monoamine oxidase A	MAOA	P21397
2	Acetylcholinesterase	ACHE	P22303
3	Beta-secretase 1	BACE1	P56817
4	Cytochrome P450 1A2	CYP1A2	P05177
5	Cytochrome P450 19A1	CYP19A1	P11511
6	MAP kinase p38 alpha	MAPK14	Q16539
7	Nitric oxide synthase, inducible	NOS2	P35228
8	Nerve growth factor receptor Trk-A	NTRK1	P04629
9	Histone deacetylase 2	HDAC2	Q92769
10	Tyrosine-protein kinase JAK2	JAK2	O60674
11	Histone deacetylase 1	HDAC1	Q13547
12	Serine/threonine-protein kinase mTOR	MTOR	P42345
13	PI3-kinase p110-beta subunit	PIK3CB	P42338
14	PI3-kinase p110-gamma subunit	PIK3CG	P48736
15	PI3-kinase p110-alpha subunit	PIK3CA	P42336
16	Phosphodiesterase 5A	PDE5A	O76074
17	Heat shock factor protein 1	HSF1	Q00613
18	Muscle glycogen synthase	GYS1	P13807
19	Protein tyrosine kinase 2 beta	PTK2B	Q14289
20	Monoamine oxidase B	MAOB	P27338
21	MAP kinase-activated protein kinase 2	MAPKAPK2	P49137
22	c-Jun N-terminal kinase 1	MAPK8	P45983
23	Protoporphyrinogen oxidase	PPOX	P50336
24	Prostanoid EP2 receptor	PTGER2	P43116
25	Butyrylcholinesterase	BCHE	P06276
26	Cannabinoid receptor 1	CNR1	P21554
27	Cyclooxygenase-1	PTGS1	P23219
28	Cyclooxygenase-2	PTGS2	P35354
29	Cannabinoid receptor 2	CNR2	P34972
30	Dual specificity mitogen-activated protein kinase kinase 1	MAP2K1	Q02750
31	Tyrosine-protein kinase JAK1	JAK1	P23458
32	Platelet activating factor receptor	PTAFR	P25105
33	Nuclear factor NF-kappa-B p105 subunit	NFKB1	P19838
34	Quinone reductase 1	NQO1	P15559
35	Estrogen receptor alpha	ESR1	P03372
36	Ribosomal protein S6 kinase 1	RPS6KB1	P23443
37	Poly [ADP-ribose] polymerase-1	PARP1	P09874
38	Intercellular adhesion molecule-1	ICAM1	P05362
39	Selectin E	SELE	P16581
40	Endothelin receptor ET-B	EDNRB	P24530
41	Endothelin receptor ET-A	EDNRA	P25101
42	Heat shock protein HSP 90-alpha	HSP90AA1	P07900
43	Arachidonate 5-lipoxygenase	ALOX5	P09917
44	Leucine-rich repeat serine/threonine-protein kinase 2	LRRK2	Q5S007
45	Androgen Receptor	AR	P10275
46	Aldo-keto-reductase family 1 member C3	AKR1C3	P42330
47	Caspase-3	CASP3	P42574
48	Caspase-9	CASP9	P55211
49	Caspase-7	CASP7	P55210
50	Myeloperoxidase	MPO	P05164
51	Cyclin-dependent kinase 2	CDK2	P24941
52	Cyclin-dependent kinase 4	CDK4	P11802
53	Xanthine dehydrogenase	XDH	P47989
54	Aldo-keto reductase family 1 member C1	AKR1C1	Q04828
55	Estrogen receptor beta	ESR2	Q92731
56	Aldose reductase	AKR1B1	P15121
57	Insulin receptor	INSR	P06213
58	Focal adhesion kinase 1	PTK2	Q05397
59	Tyrosine-protein kinase TIE-2	TEK	Q02763
60	Heat shock 70 kDa protein 1	HSPA1A	P0DMV8
61	Tyrosine-protein kinase Lyn (by homology)	LYN	P07948

**Table 4:** Binding energy between Q-Markers and core targets.

Target	Binding Energy (kcal/mol)			
	Imperatorin	Oxypeucedanin	Psoralen	Bergapten
CASP3	−7.3	−5.0	−6.6	−6.5
ESR1	−7.3	−5.5	−6.3	−6.8
HSP90AA1	−7.7	−5.3	−4.7	−5.5
MTOR	−7.4	−6.5	−6.4	−6.3
MAPK8	−6.1	−5.2	−5.6	−5.9

## Data Availability

The data presented in this study are available on request from the corresponding author.

## Sample Availability

Samples of the plant materials of *A. dahurica* are available from the authors.
